# Improved technique for order of preference by similarity to ideal solution method for identifying key terrain in cyberspace asset layer

**DOI:** 10.1371/journal.pone.0288293

**Published:** 2023-07-13

**Authors:** Longhui Liu, Yang Zhou, Qing Xu, Qunshan Shi, Xiaofei Hu

**Affiliations:** 1 PLA Strategic Support Force Information Engineering University, Institute of Geospatial Information, Zhengzhou, Henan, China; 2 Key Laboratory of Spatiotemporal Perception and Intelligent Processing, Ministry of Natural Resources, Zhengzhou, China; 3 Collaborative Innovation Center of Geo-Information Technology for Smart Central Plains, Zhengzhou, China; Vinnytsia National Technical University, UKRAINE

## Abstract

Reinforcing weak cyberspace assets is an urgent requirement to defend national cybersecurity. Cyberspace key terrain (CKT) is a theory recently proposed for sensing cyberspace posture. Identifying CKT in the asset layer is essential for supporting cyberspace defense decisions. Existing methods ignore the influence of the multi-attribute correlation of cyberspace nodes and cyber attack mission (CAM) diversity, which restricts the recognition accuracy of CKT. To improve the accuracy of CKT identification and explore the relationship between CKT and CAM, we propose an improved cosine similarity technique for order of preference by similarity to the ideal solution (CosS-TOPSIS) method to model CKT and construct a CAM based on the MITRE adversarial tactics, techniques, and common knowledge (ATT&CK) framework to examine the influence of different weighted CAM on modeling CKT. Based on the vulnerability value calculation method of the cyber system in the common vulnerability scoring system version 3.1 (CVSS 3.1), we evaluated the effectiveness of CosS-TOPSIS in identifying CKT using three metrics: correlation coefficient, root mean square error, and mean absolute error. Our experiments showed that, in comparison with the TOPSIS method, the accuracy of the proposed method for identifying CKT improved by 8.9%, and the root mean square error reduced by 16%; simultaneously, CAM was proven to be an essential factor in identifying CKT. The feasibility and reliability of CosS-TOPSIS in identifying CKT and the close relationship between CAM and CKT identification were demonstrated experimentally. In our work, we utilized cosine similarity and FAHP to improve the baseline method. We also introduced three indicators to evaluate the method’s reliability. Drawing from ATT&CK, we recommend CAM as a tool for sensing changes in the cyberspace environment and explore its relationship with CKT. Our work has great application potential for identifying cyberspace vulnerabilities, supporting cyberspace defense, and securing national cyberspace facilities.

## Introduction

Cyberspace, the fifth dimension of human activity [1], has broken the spatiotemporal boundaries of traditional space and constructed new fields of human activity. However, the general vulnerability of cyberspace [2] presents increasingly severe security risks for enterprises and countries. For example, Ukraine [3] and Georgia [4] suffered large-scale cyberattacks that severely affected economic development and social order. Cyberspace surveying and mapping (CSM) is a new theoretical and technical field for securing national cyber security [5] and an effective means for conducting extensive data mining and applications in cyberspace [6]. Identifying the key terrain in cyberspace, locating cyberspace weaknesses, and improving cyberspace situational awareness and defense capabilities are important research topics in CSM.

The terrain concept originates from geography and refers to the general term for various undulating forms of the Earth’s surface [7]. Scholars have introduced the concept of "terrain" in the study of cyberspace to describe its hierarchical structure [8, 9]. Similar to geospatial highlands, “cyberspace-critical terrain” is used to describe network connections and cyberspace nodes that are critical to both friendly and enemy forces [10, 11]. These nodes are perceived as network IPs. Currently, the commonly used methods for modeling and analyzing critical cyberspace terrain are the observation and fields of fire, cover and concealment, obstacles, key terrain, and avenues of approach (OCOKA) method [12], directed graph method [13], and TOPSIS method [14].

However, current methods for identifying key terrain in cyberspace ignore the correlation between various attributes of nodes and need to focus on the impact of cyberattack tasks on CKT, which increases the challenge of locating and identifying weaknesses in cyberspace.

To solve the problem of insufficient CKT identification accuracy, we propose the cosine similarity technique for order of preference by similarity to ideal solution (CosS-TOPSIS) based on cosine similarity improvement to identify the key terrain in cyberspace at the asset layer. We introduce a cyberspace attack mission from the perspective of hackers and analyze its impact on identifying the key terrain in cyberspace.

This study makes the following contributions: First, a CosS-TOPSIS-based modeling method is proposed for CKT. We experimentally demonstrated that the proposed method could reduce the influence of the correlation between multiple attributes of network nodes in identifying the critical terrain in cyberspace and improve its accuracy. Second, based on the MITRE (A security research institute) adversarial tactics, techniques, and common knowledge (ATT&CK) framework, we proposed cyber-attack tasks and applied the fuzzy analytic hierarchy process (FAHP) to calculate their weights and extend the dynamics of CKT to examine the impact of different weighted cyber-attack tasks on identifying key terrain in cyberspace.

The remainder of the paper is organized as follows: Section 2 presents the current research methods for identifying CKT, Section 3 introduces the cyber-attack task, Section 4 presents the FAHP and CosS-TOPSIS methods for constructing analytic hierarchy process models, Section 5 explains the composition of the network terrain of power secondary information systems and criterion layer in the fuzzy hierarchical analysis model, Section 6 analyzes the experimental results and discusses the impact of the cyber-attack task on CKT identification, and finally, Section 7 summarizes the study findings.

## Related work

Hobbs [12] first applied the OCOKA method (most commonly used in traditional military terrain analysis) to cyberspace by applying concepts such as line of sight and field of fire, concealment and cover, and obstacles and pathways. Argauer et al. [13] modeled the cyber terrain in terms of directed graphs to identify possible targets of cyber attacks, which referred to the relationship between services and privileges associated with hosts in capturing them. However, they ignored other elements such as software, which constituted the terrain of cyberspace. Raymond et al. [8] extended and applied the OCOKA method based on Hobbs’ analysis and proposed the framework and steps for critical terrain analysis in cyberspace, i.e., identifying potential targets, considering the visual field and projection range of current technology first, then listing the entry paths and obstacles of the targets, and finally determining the key terrain through iterative analysis.

Endsley [15] initially proposed a human decision model of situational awareness and a three-level model of situational element perception, understanding, and prediction, which has been one of the most widely used international situational awareness models. MITRE [16] reinterpreted Endsley’s three-level model as a framework for the network field, which included network awareness (asset and configuration management), threat awareness (identifying events and suspicious behaviors), and task awareness (critical dependence, real-time response, risk assessment, and informed defense plans). Jajodia et al. [17] reinforced the work of MITRE and Endsley by stating that arguing that understanding the vulnerabilities, interdependencies, and supporting tasks of cyber systems is fundamental to protecting cyber infrastructures and missions. Jingmin [18] proposed an integrated cyber-physical situational management framework and mission situational management process; they work in tandem to adapt to task and network terrain changes, thereby enabling network situational awareness. Jakobson [9] proposed the concept of resilient tasks and discussed the interplay between them and IT infrastructure. Pethon [14] first applied TOPSIS to CKT detection to identify U.S. Navy industrial control system assets; they studied and analyzed the actual weights affecting the critical cyber terrain to achieve tactical-level situational awareness, thus mapping the critical terrain for cyber situational awareness.

The analytic hierarchy process (AHP) is a combined qualitative and quantitative system analysis method proposed by Saaty, an operations research scientist at the University of Pittsburgh in the 1970s [19]. However, the consistency test of the judgment matrix in this method requires several adjustments to the judgment matrix elements, which requires significant work. To address this problem, scholars proposed the widely used fuzzy analytic hierarchy process (FAHP) [20].

The TOPSIS method is a scientifically based multi-attribute decision-making approach for evaluating goals using multidimensional attributes [21]. However, its reliance on calculating the Euclidean distances between positive and negative solutions overlooks the inherent multidimensional correlations in cyberspace systems. Moreover, assuming attribute independence by default reduces the relevance of the resulting associations. Attributes with strong correlations can be treated as one-dimensional, resulting insignificant interference among multidimensional attributes. This interference substantially undermines the validity of the original Euclidean distances between ideal states. When dealing with cyberspace systems, it is crucial to fully consider the correlations among various CTK attributes. Thus, it becomes essential to maintain a strong relationship between CAM and CKT to avoid the adverse effects of multicollinearities. Failing to do so would raise doubts about the reliability and accuracy of identifying CKT vulnerabilities and their respective locations.

Scholars improved the TOPSIS method by replacing the Euclidean distance with the Mahalanobis distance [22, 23] to compensate for the shortcomings of the ideal solution ranking method. However, scholars have not discussed the differences between these methods in terms of CKT modeling.

In general, all the mission considerations in CKT identification methods are for purpose of warfare, lacking a cyberattack mission framework that describes the attack purpose itself. Most methods of CKT detection draw qualitative conclusions from cyberspace terrain analysis and lack quantitative analysis, thus making it challenging to support cybersecurity defense decisions. The TOPSIS method proposed by Pethon ignores the AHP method, the problems of correlation between multiple attributes in the criterion layer, and the difficulty in constructing a consistent judgment matrix for multi-attribute hierarchical variants. This indicates that constructing a critical terrain detection algorithm that quantitatively describes the cyberspace asset layer associated with cyberspace attack missions remains a challenge.

To address the above problems, this paper proposes a framework for describing cyberspace attack missions based on a hacker’s perspective and adopts an ideal solution ranking method based on cosine similarity improvement to reduce the impact caused by indicator correlation in the traditional TOPSIS method. When constructing the hierarchical analysis model, the FAHP method is used instead to quickly construct a fuzzy consistency judgment matrix.

## Cyberspace attack mission

The ATT&CK framework was proposed by the MITRE Institute at the Massachusetts Institute of Technology Computer Laboratory to describe the path of a cyberspace attack from the attacker’s perspective. It contains 14 tactics and 177 techniques and 348 sub-techniques for each phase of a cyber attack [24]. The tactical compositions are listed in Table 1.

**Table 1:** Adversarial tactics, techniques, and sub-technique framework.

Tactic	Tactical combination
Reconnaissance	Collect information for future attacks.
Resource development	Obtain resources needed for attacker actions.
Initial access	Enter the target network and establish a foothold.
Execution	Run malicious code.
Persistence	Maintain foothold for attacks and gain permanent control.
Privilege escalation	Obtain the highest network authority.
Defense evasion	Avoid discovery.
Credential access	Steal account numbers, passwords, vouchers, etc.
Discovery	Discover target’s network environment.
Lateral movement	Roam the intranet to attack other networks.
Collection	Collect data of interest.
Command and control	Communicate with and manipulate the target network.
Exfiltration	Export stolen data.
Impact	Interrupt/damage/destroy the target’s network and data.

A CAM should describe the effect achieved by an attack based on the method of the cyber attack instead of the mission purpose [9, 18, 25]. ATT&CK is an old framework for classifying cyber attack techniques, dividing them into 14 categories. However, hackers can use all 14 tactics simultaneously when conducting cyber attacks. A CAM can involve multiple tactics in any combination. Therefore, the 14 tactics in the ATT&CK framework need to be reclassified.

**Fig 1:**
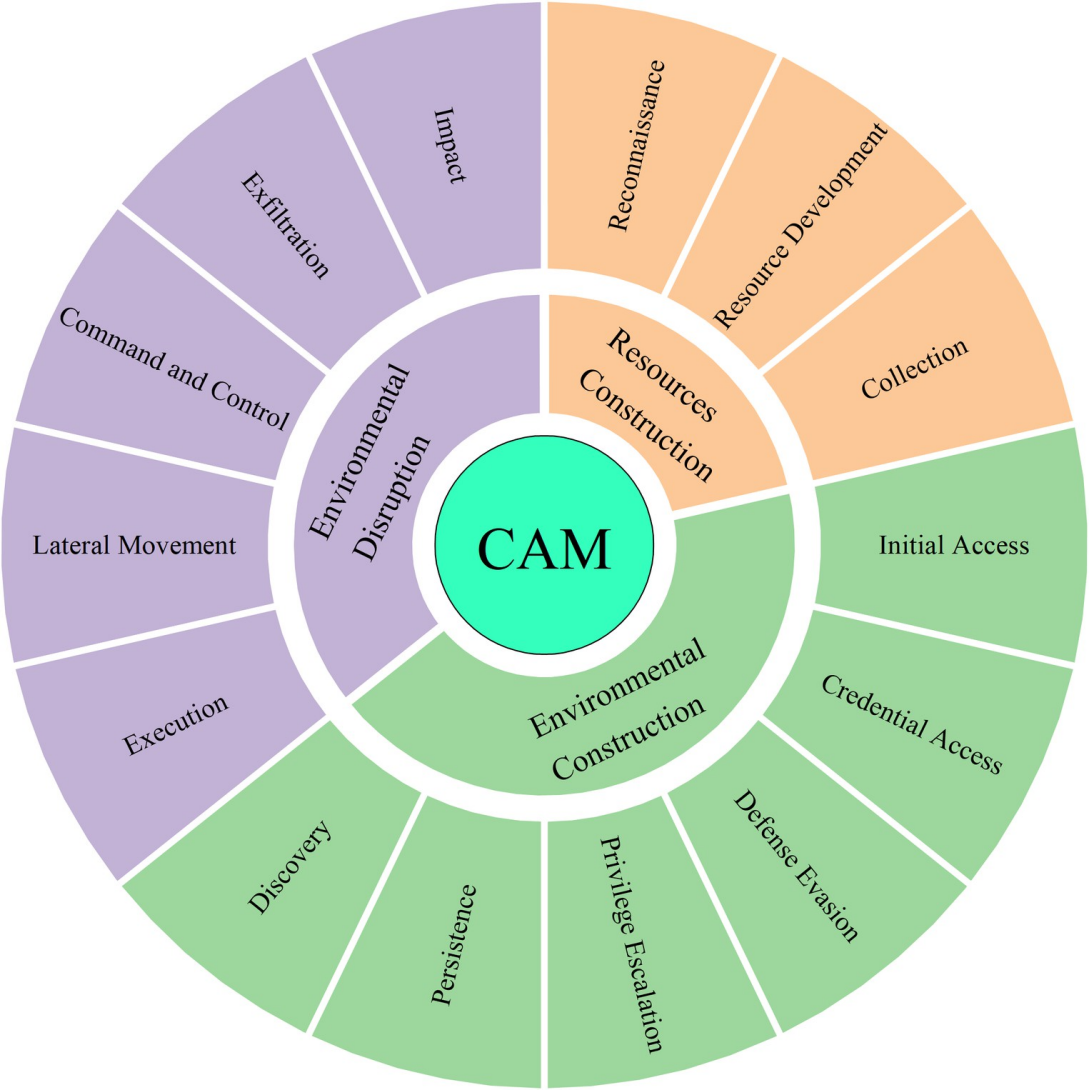
Relationship between cyberattack missions (CAM) and tactics. Three CAM categories are distinguished by different colors and mapped to the corresponding ATT&CK tactics.

In our study, the 14 tactics were divided into three major categories based on the impact of cyberattacks: resource construction, environment construction, and environmental disruption, as shown in Fig 1. Resource building, i.e., accessing the attacked party’s information and resources, includes three tactics: reconnaissance, resource development, and collection. Environment building, i.e., identifying the adversary’s network environment, obtaining system privileges, and hiding identity, includes six tactics: initial access, persistence, permission enhancement, defense bypass, credential acquisition, and discovery. Environmental disruption, i.e., exporting data from the adversary’s network or destroying the adversary’s network environment, includes five tactics: execution, lateral movement, command and control, data exfiltration, and impact.

Managers and decision makers assign CAMs to technicians without requiring in-depth knowledge of the underlying technologies involved in task completion. CAM encompasses 14 tactics within the ATT & CK framework, each encompassing various cyberattack techniques. These tactics serve not only as CAM components but also as a system for classifying cyber attack technologies. Therefore, CAM provides cyber security defense support for cyberspace management and decision-makers, enabling them to adopt a hacker’s perspective. A cyberspace attack mission combines one or more tactics into one of the three major categories. Six CAM examples that hackers may develop are listed in Table 2.

**Table 2:** Six hacker mission examples.

Symbol	Tactical combination
M1	Reconnaissance + Privilege escalation + Collection + Exfiltration
M2	Resource development + Execution + Defense evasion + Impact
M3	Reconnaissance + Initial access + Defense evasion + Exfiltration
M4	Reconnaissance + Persistence + Credential access + Command and control
M5	Initial Access + Privilege escalation + Collection + Impact
M6	Reconnaissance + Discovery + Lateral movement + Impact

## Methodology

**Fig 2:**
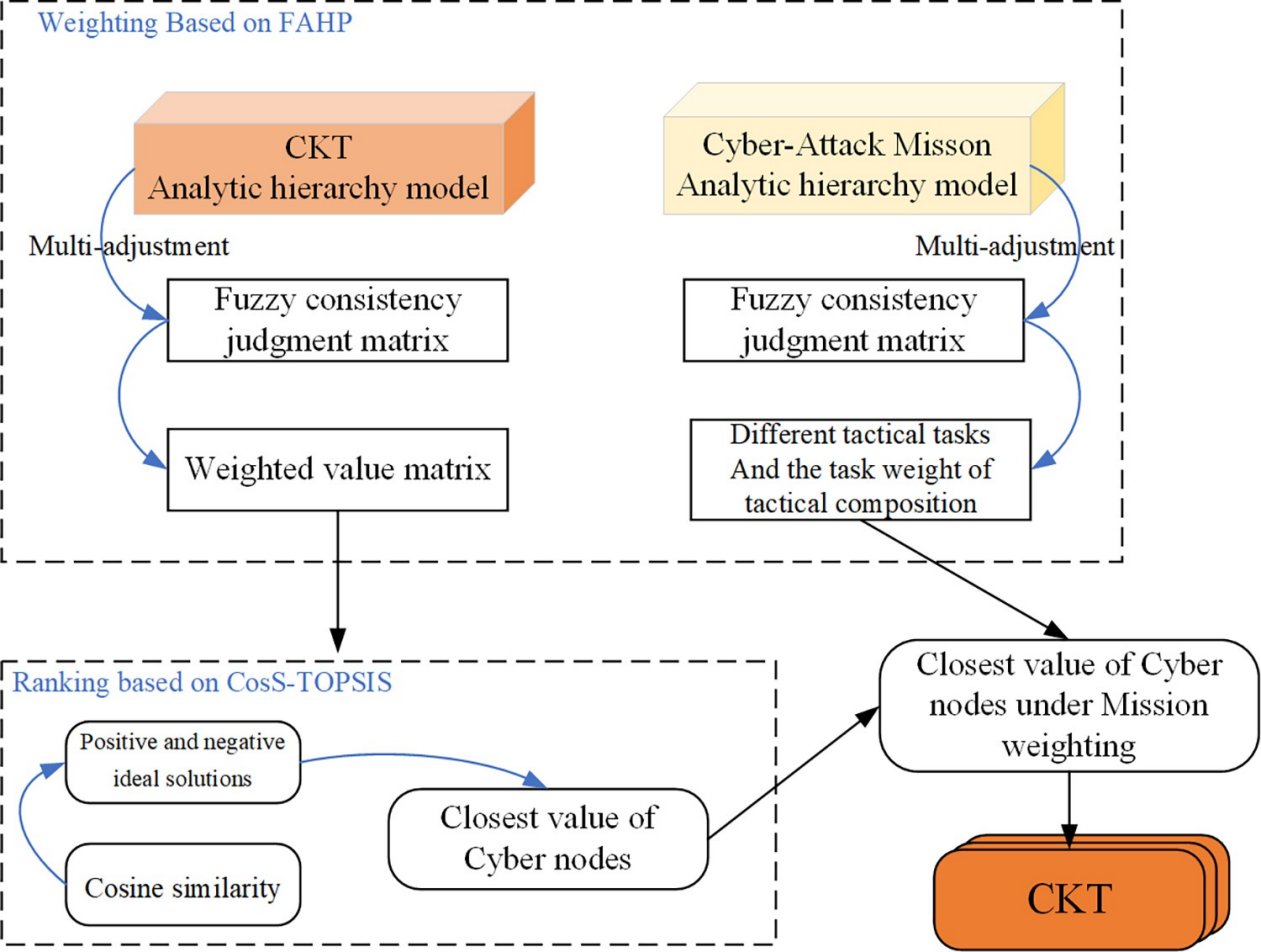
Technical cosine similarity Technique for Order-of-Preference by Similarity (TOPSIS) to the Ideal Solution implementation with weighting and sequencing processes. The final determination of cyberspace key terrain is dependent on the weight assigned to each cyberattack mission. The correction process incorporates the weights and takes into account the impact of each mission. In this context, CosS (cosine similarity) is utilized as a metric.

The multi-attribute decision-based modeling of CKT must address both the weighting and ranking of problems. In terms of weighting, we constructed a fuzzy consistency judgment matrix based on the FAHP and rapidly adjusted the elements of the fuzzy matrix to make it consistent [26]. In terms of ranking, we replaced the Euclidean distance with cosine similarity and considered that the smaller the angle of the pinch, the more significant the similarity would be (i.e., closer to the ideal solution), and corrected the cosine function with a logarithmic function to make the cosine similarity a better alternative to the Euclidean distance [27]. The technical route of the proposed method is shown in Fig 2.

This section presents the principles for constructing hierarchical analysis models for critical terrains in cyberspace and attack missions. It then introduces the principles of the CosS-TOPSIS method for solving the weighted closest value of each network node.

### FAHP model

**Fig 3:**
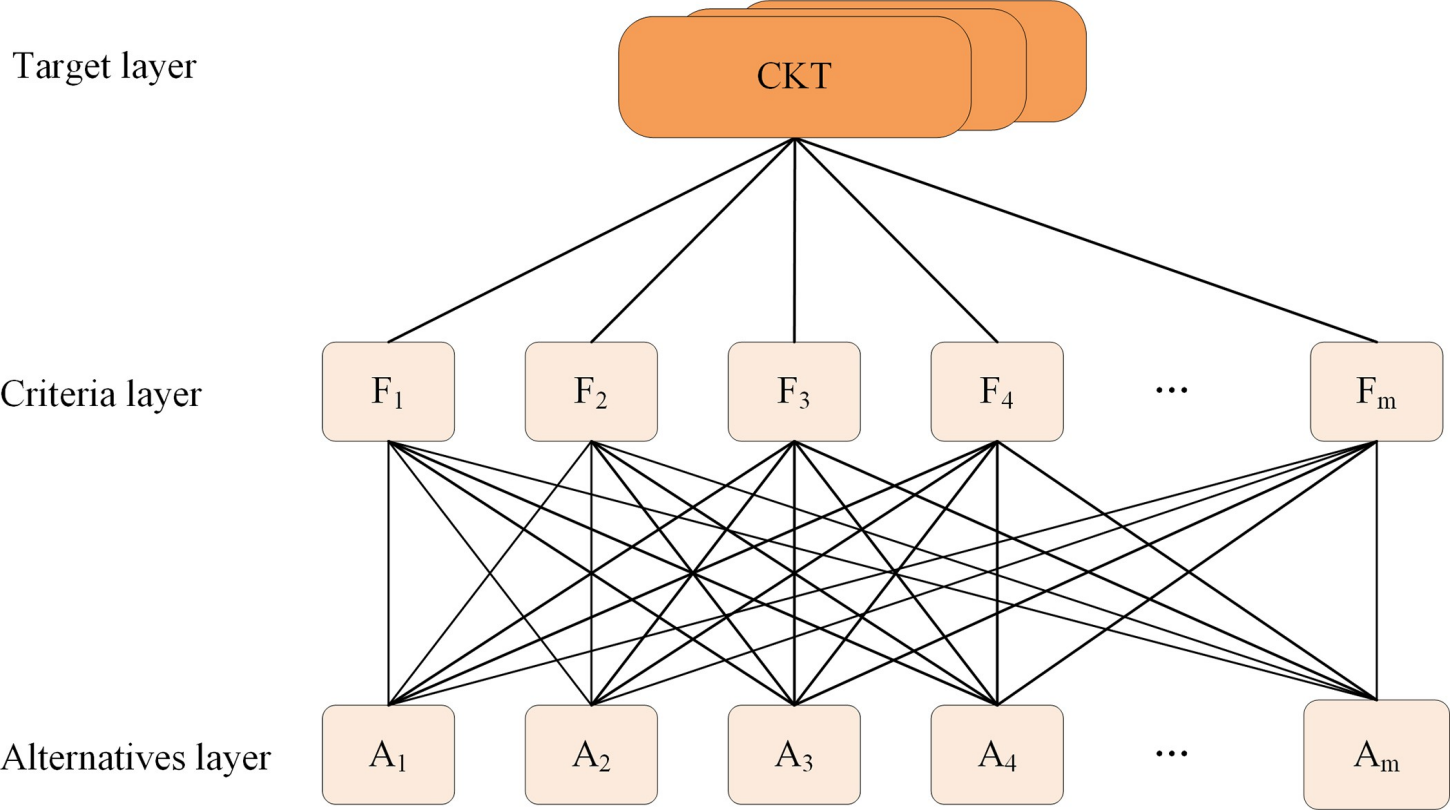
Fuzzy analytic hierarchy process-based hierarchical analysis model. The criterion layer serves as the evaluation standard, while the target layer is derived from the alternative layer through filtering. CKT refers to cyberspace key terrain.

A hierarchical model was established for CKT (shown in Fig 3). The first layer is the target layer, i.e., the cyberspace key terrain; the second layer is the criterion layer, which contains the characteristic properties of the asset layer of cyberspace terrain, denoted as *F*_ℓ_, ℓ∈{1,…,*k*}; the third layer is the alternative layer, which refers to the connected nodes in the cyberspace and is denoted by *A*_*m*_, *m*∈{1,…,*n*}.

The CAM hierarchy model was constructed in a manner similar to the CKT hierarchy analysis model; it has a criterion layer describing the network information security attributes of information transmission and an alternative layer of 14 tactics in the ATT&CK framework.

FAHP, like AHP, allows "all-important tangible and intangible, quantitative, and qualitative factors" to be included and measured [26]. In our work, the criterion layer *F*_ℓ_ of the hierarchical analysis model was evaluated by experts familiar with cyberspace and experienced in cyber attacks. The fuzzy judgment scale for the evaluation index is listed in Table 3.

**Table 3:** Fuzzy judgment scale elements.

Scale	Meaning
0.5	The two assets are equally important.
0.6	One is slightly more important than the other.
0.7	One is obviously more important than the other.
0.8	One is much more important than the other.
0.9	One is more important than the other.

*Note*: 0.1, 0.2, 0.3, and 0.4 are anti-comparisons. If the comparison value of assets *i* and *j* is *b*_*ij*_, the comparison value of assets *j* and *i* is *b*_*ji*_ = 1−*b*_*ij*_.

The fuzzy matrix constituting the asset layer of CKT is $\tilde{R}$.

$$\tilde{R}=[\begin{array}{cccc} r_{11} & r_{12} & \cdots & r_{1m}\\ r_{21} & r_{22} & \cdots & r_{2m}\\ \cdots & \cdots & \ddots & \cdots \\ r_{n1} & r_{n2} & \cdots & r_{nm} \end{array}],$$

$$r_{ij}=\frac{1}{K}(r_{ij}^{\left(1\right)}+r_{ij}^{\left(2\right)}+\cdots +r_{ij}^{\left(k\right)}),$$ (1)

where *i* = {1,2,⋯,*n*}, *j* = {1,2,⋯,*n*}, and $r_{ij}^{\left(k\right)}$ denotes the evaluation value of the *k*^th^ expert on the criterion layer of the network asset *A*_*i*_. $\tilde{R}$ has the following properties: *r*_*ii*_ = 0.5, *r*_*ij*_ = 1−*r*_*ji*_, and *r*_*ij*_ = *r*_*it*_−*r*_*jt*_, *t* = {1,2,⋯,*n*}.

Subsequently, the fuzzy matrix $\tilde{R}$ was adjusted for consistency. From the fuzzy matrix, to select the highest confidence in a row of judgments (*r*_11_, *r*_12_,⋯,*r*_1*m*_) with the row and matrix $\tilde{R}$ in each row to perform subtraction, if the number of differences obtained *n* are constant, then we do not need to adjust; otherwise, we need to adjust to the subtracted row so that the two *n* differences are constant, the adjusted fuzzy consistency matrix for *R*. The process of constructing the fuzzy consistency judgment matrix for a cyberspace attack mission is similar to that of the CKT asset layer.

The weights of the fuzzy consistency matrix *R* obtained by a two-by-two comparison of the cyber information security attributes of the information transmission related to the cyber-attack task are denoted by *W*_*α*1_,…,*W*_*αk*_, where *W*_*αk*_ is the weight of the cyber information security attribute *F*_*ℓ*_ that affects information transmission. The weight assigned to the characteristic attributes of the topography of the asset layer network space is denoted by *W*_*β*1_,…,*W*_*βk*_, which is given by the FAHP calculation.

Based on the fuzzy consistency matrix, the fuzzy weights can be obtained using the geometric mean method with the following formula:

$$W_{\alpha i}=r_{i}\times {\left(r_{1}+r_{2}+\cdots +r_{n}\right)}^{-1},$$ (2)

$$r_{i}={\left(a_{i1}\times \cdots {\times a}_{ij}{\times a}_{in}\right)}^{\frac{1}{n}},$$ (3)

where *i*,*j* = 1,2,⋯,*n*; the weights *W*_*βk*_ of the feature attributes of the CKT asset layer can be derived in the same way.

### Alternative calculation

The weighted value matrix *V* is obtained by multiplying the fuzzy normalized decision matrix *R* with the weights *W*_*βk*_ of the characteristic attributes of *t*. The CKT asset layer is expressed as follows:

$$V=[\begin{array}{cccc} v_{11} & v_{12} & \cdots & v_{1m}\\ v_{21} & v_{22} & \cdots & v_{2m}\\ \cdots & \cdots & \ddots & \cdots \\ v_{n1} & v_{n2} & \cdots & v_{nm} \end{array}]=RW_{\beta k}=[\begin{array}{cccc} r_{11}w_{1} & r_{12}w_{2} & \cdots & r_{1m}w_{n}\\ r_{21}w_{1} & r_{22}w_{2} & \cdots & r_{2m}w_{n}\\ \cdots & \cdots & \ddots & \cdots \\ r_{n1}w_{1} & r_{n2}w_{2} & \cdots & r_{nm}w_{n} \end{array}].$$ (4)

The positive ideal solution is the set of highest-scoring elements in the weighted value matrix *V*, $C^{+}=\{c_{1}^{+},c_{2}^{+},\cdots ,c_{m}^{+}\}$; the negative ideal solution is the set of lowest-scoring elements in the weighted value matrix *V*, $C^{-}=\{c_{1}^{-},c_{2}^{-},\cdots ,c_{m}^{-}\}$.


$$c_{j}^{+}=\max\left\{v_{ij}|1\leq i\leq n\right\}$$ (5)

$$c_{j}^{-}=\min\left\{v_{ij}|1\leq i\leq n\right\}$$ (6)

The CosS-TOPSIS method converts the Euclidean distance calculation in the traditional TOPSIS method into a calculation of the cosine angle in vector space, which can reduce the correlation between cyberspace assets [27].

First, in an *n*-dimensional vector space, the equation for the cosine of the angle between vectors, $\vec{A}={\left[x_{1},x_{2},\cdots ,x_{n}\right]}^{T}$ and $\vec{B}={\left[y_{1},y_{2},\cdots ,y_{n}\right]}^{T}$, is expressed as follows:

$$\cos\left(\theta \right)=sim(x_{i},y_{i})=\frac{\sum_{i=1}^{n}x_{i}\times y_{i}}{\sqrt{\sum_{i=1}^{n}{\left(x_{i}\right)}^{2}}\times \sqrt{\sum_{i=1}^{n}{\left(y_{i}\right)}^{2}}}$$ (7)

Then, the logarithmic function with a base of 0.5 and the power of the cosine function are chosen to correct the cosine similarity. This composite function is a decreasing function, and its meaning is expressed as follows: the more significant the similarity, the smaller the distance will be. The formulae for calculating each cyberspace asset *A*_*i*_ for positive and negative ideal solutions using the composite function are expressed as follows:

$$d(A_{i},C^{+})=\log_{\frac{1}{2}}(\frac{\mathrm{sim}(v_{i},c^{+})+1}{2}),$$ (8)

$$d(A_{i},C^{-})=\log_{\frac{1}{2}}(\frac{\mathrm{sim}(v_{i},c^{-})+1}{2}).$$ (9)

Finally, the average of each indicator for the positive and negative ideal solutions is calculated; then, the average is subtracted in all dimensions to amplify the gap between scenarios using the following equations.


$$d(A_{i},C^{+})=\log_{\frac{1}{2}}(\frac{\mathrm{sim}(v_{i}-c^{\mathrm{avg}},c^{+}-c^{\mathrm{avg}})+1}{2})$$ (10)

$$d(A_{i},C^{-})=\log_{\frac{1}{2}}(\frac{\mathrm{sim}(v_{i}-c^{\mathrm{avg}},c^{-}-c^{\mathrm{avg}})+1}{2})$$ (11)

Closeness *f*_*i*_ refers to the closeness between each alternative and the ideal solution; the smaller the closeness, the further away the alternative is from the ideal solution, expressed as follows:

$$f_{i}=\frac{d(A_{i},C^{-})}{d(A_{i},C^{-})+d(A_{i},C^{+})}.$$ (12)

### Calculation of closest value in CAM-weighting

The CAM weights are denoted by *W*_*α*1_,…,*W*_*αk*_, and closeness is denoted as *f*_1_,…,*f*_*i*_; then, the CKT asset layer can be expressed as

$$P_{k,i}=[\begin{array}{ccc} W_{\alpha 1}f_{1} & \cdots & W_{\alpha k}f_{1}\\ \vdots & \ddots & \vdots \\ W_{\alpha 1}f_{i} & \cdots & W_{\alpha k}f_{i} \end{array}].$$ (13)

## Case study

### Cyberspace asset layer

**Fig 4:**
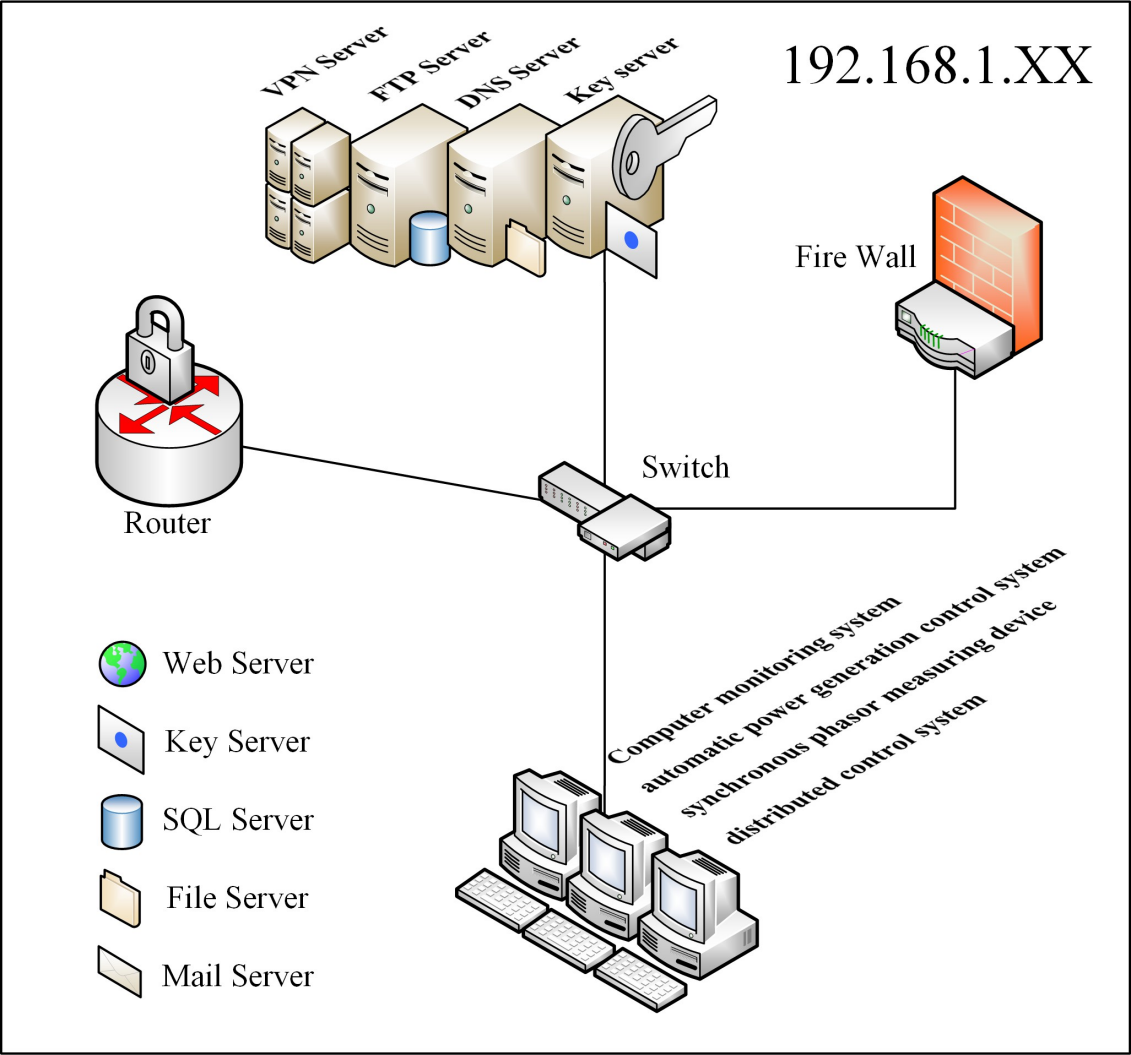
Cyberspace asset layer, including hardware devices and virtual services. The network node in this case has IP addresses within the 192.168.1.x private subdomain.

The asset layer of cyberspace comprises the physical and logical layers. The physical layer comprises all the routers, switches, servers, and workstations, while the logical layer comprises all the virtual ports, UDP and TCP protocols, office automation systems running on workstations, management information systems, RPC, IMAP, DNS, and HTTP services. The network nodes (IPs) of this cyberspace terrain comprise different devices, transport protocols, and software services. As shown in Fig 4, the cyberspace nodes with an IP address of 192.168.1. XX consists of servers, computers, switches, routers, VPN servers, FTP servers, DNS servers, and automatic power monitoring systems.

Utilizing web-crawler technologies and the ZoomEye cyberspace mapping engine, we obtained the network nodes within a specific area. The tool identified 20 cyber nodes, providing details such as IP addresses, autonomous domain organization, asset categories, components, ports, vulnerabilities, and other attributes. Table 4 presents a sample of electric power-related node information.

**Table 4:** Sample electric power secondary information system nodes.

Symbol	Cyberspace node	Vulnerability value
A1	111.70.12.221 (ChangDe Substation)	111.0
A2	111.70.12.47 (DongLin Substation)	111.0
A3	221.120.40.146 (HuKe Substation)	203.4
A4	111.70.0.241 (LongEn Substation)	84.4
A5	221.120.43.214 (NanTou Substation)	137.9
A6	111.70.1.234 (SongShan Substation)	264.5
A7	111.70.12.220 (Tiashan Substation)	111.0
A8	111.70.15.50 (WoLong Substation)	401.7
A9	220.130.135.176 (LiuLang Substation)	29.3
A10	221.120.49.56 (ZhongLi Substation)	321.4
A11	111.70.15.240 (Eastern Power Plant)	99.5
A12	111.70.15.206 (Eastern Power Plant)	244.1
A13	220.132.72.117 (Guishan Power Plant)	494.4
A14	111.70.12.104 (Guishan Power Plant)	166.8
A15	111.70.0.70 (Guishan Power Plant)	29.3
A16	60.248.159.104 (Guishan Power Plant)	29.3
A17	111.70.0.163 (Guishan Power Plant)	111.0
A18	111.70.0.217 (Guishan Power Plant)	600.3
A19	59.124.225.19 (Guishan Power Plant)	248.2
A20	61.219.219.81 (Guishan Power Plant)	347.9

*Notes*: The vulnerability values in the table are obtained by the CVSS method, and the calculation formula is as shown in Formula 14.

### Construction of target layer

The application of FAHP necessitates the construction of a criterion layer, which is a guideline for experts or expert systems to evaluate alternatives. To construct the hierarchical analysis model for a cyberspace attack mission, we used the network information security attributes proposed by the U.S. Cybersecurity and Infrastructure Agency (CISA) to construct the criterion layer [28]. We used confidentiality, integrity, availability, controllability, and non-repudiation, as the five attributes of the cyberspace attack mission to construct the fuzzy consistency judgment matrix. Confidentiality refers to the information in the network being protected from unauthorized entities. Integrity refers to the property that information remains unmodified, uncorrupted, and not lost during storage or transmission. Availability refers to the ability of authorized entities or users to access and use the information as required. Controllability refers to the ability of legitimate users to control the information, and non-repudiation refers to the maintenance of authenticity on both sides of an information exchange.

When constructing a hierarchical analysis model of CKT, we consider the components, hardware devices, ports, network protocols, and operating systems of IP as the characteristic attributes of the physical layer of cyberspace and construct the guideline layer accordingly. The components refer to the applications, support, and services detectable by the IP; the hardware devices refer to all hardware devices under IP, including but not limited to routers, switches, workstations, and industrial computers. Ports are outlets for communication between devices and the outside world; in this paper we refer to virtual ports. The network protocols refer to the set of rules, standards, or conventions established for data exchange; the operating system refers to the interrelated system and software programs that control computer operations, employ and run hardware and software resources, and provide public services to organize user interactions.

**Fig 5:**
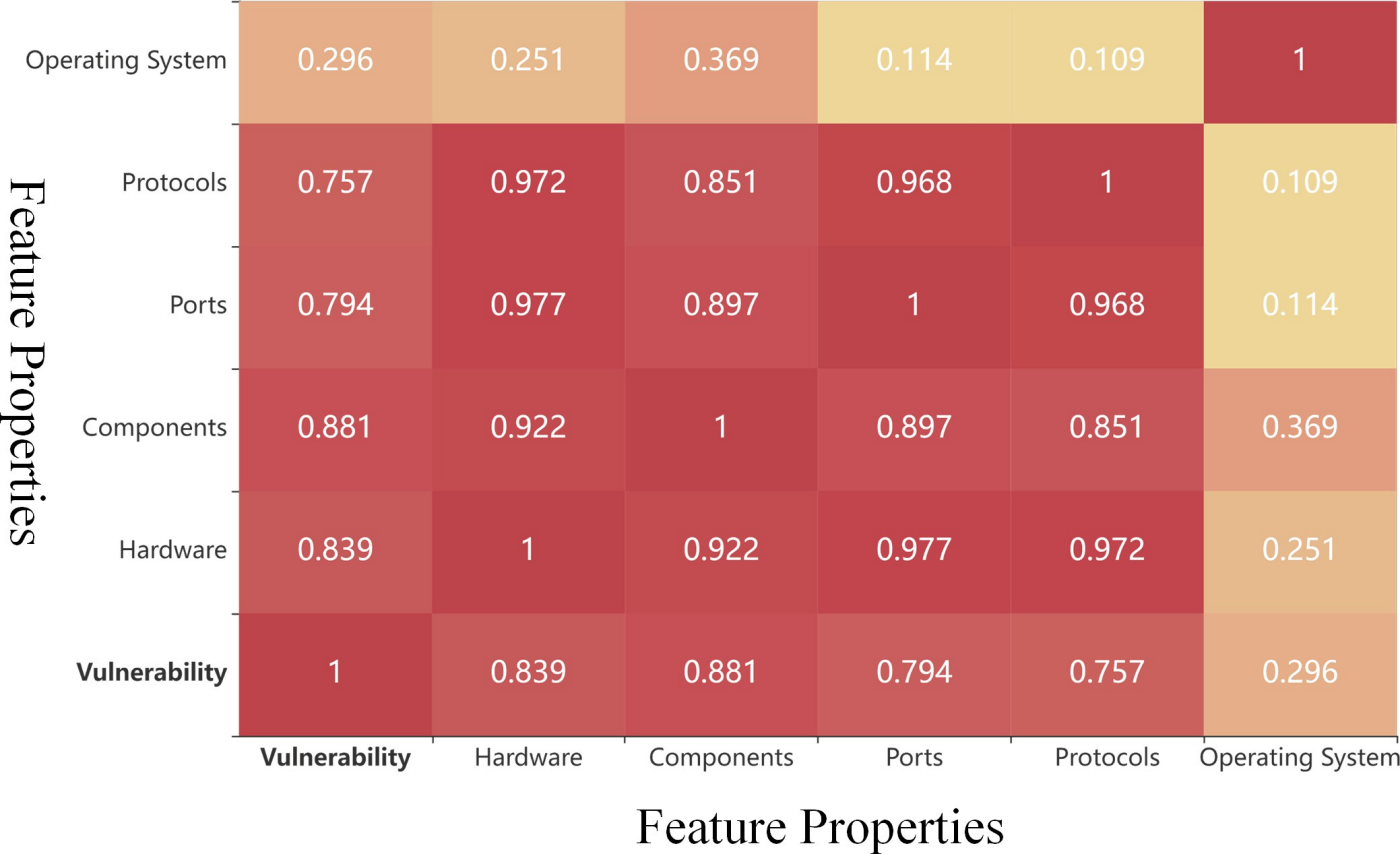
Correlation between feature attributes. The color becomes redder as the correlation strengthens. The Spearman rank correlation coefficient method was used to determine the relationship between attributes of the cyber node.

The correlation between each attribute in the hierarchical analysis model of CKT is shown in Fig 5. The vulnerability value has a high correlation with components, hardware devices, ports, and network protocols, and a low correlation with the operating system. There are also strong correlations among hardware devices, ports, network protocols, and components. Therefore, the correlation between attributes must be considered when identifying CKT.

### Calculation of vulnerability values for cyber systems based on the common vulnerability scoring system (CVSS) 3.1

The attributes of cyberspace nodes detected through the cyberspace engine are often positively correlated with the vulnerability within that node, i.e., the more the assets, the more the loopholes. Therefore, several attributes were positively correlated with vulnerability when the criterion layer was constructed.

In this study, we evaluated the effectiveness of CosS-TOPSIS by considering the "criticality equals destructiveness" methodology of systems science [29–31] and the strong positive correlation between the metrics in the criterion layer and vulnerability of the cyber system, using the vulnerability value of the cyber system as an experimental control group.

CVSS is an open standard for characterizing vulnerabilities and their severity [32]. CVSS focuses on the attributes of the vulnerability itself and can accurately assess the vulnerability value of a single asset [33, 34]. However, our study focuses on nodes (IPs) in cyberspace, where an IP may contain multiple network devices, software, and hardware with *n* loopholes. Therefore, we used the cumulative method to determine the vulnerability value of an IP, i.e., the vulnerability scores of multiple devices under a specific IP were cumulated as the vulnerability value of that IP. A total of *t* device vulnerabilities are detected in *n* devices under a certain IP, and the value score of the vulnerabilities is *M*_*i*_ for *i*∈{1,⋯,*n*}; then, the vulnerability value of the IP is

$$Val=\sum_{1}^{t}M_{i}.$$ (14)

## Results and discussion

### Accuracy evaluation

#### Spearman’s rank correlation coefficient (SRC)

SRC is a commonly used measure of the degree of correlation between hierarchically ordered variables [35]. For a sample size of *n*, the formula for calculating the correlation coefficient, *ρ*, is as follows:

$$\rho =\frac{\frac{1}{n}\sum_{i=1}^{n}\left(x_{i}-\bar{x})\times (y_{i}-\bar{y}\right)}{\sqrt{\left({\frac{1}{n}\sum_{i=1}^{n}\left(x_{i}-\bar{x}\right)}^{2})\times ({\frac{1}{n}\sum_{i=1}^{n}\left(y_{i}-\bar{y}\right)}^{2}\right)}},$$ (15)

where *x*_*i*_ and *y*_*i*_ represent the ranks between sample data, and $\bar{y}$ and $\bar{x}$ represent the average rank.

Both TOPSIS and improved TOPSIS require calculating the closest fair value, which is also a data point in terms of rank; therefore, it is appropriate to use SRC to calculate the correlation between each method and the fragility value of the cyber system. The higher the correlation coefficient between each method and the network system fragility value calculated based on CVSS 3.1, the better the method is. Considering SRC as the accuracy of identifying the CKT asset, the accuracy of each method can be calculated as follows:

$$Accuracy=\frac{V-V_{b}}{1}\times 100\%,$$ (16)

where *V* represents the SRC of our method, and *V*_*b*_ represents the SRC of the contemporary TOPSIS method.

#### Root mean square error (RMSE) and mean absolute error (MAE)

RMSE and MAE are measures of deviation in the observed value from the actual value [36], and they have a wide range of applications in the fields of surveying, mapping, and remote sensing. The calculation principles are shown in Eqs 16 and 17. Although RMSE typically yields higher values than MAE, it is widely used. Hence, both of these indicators were used for the calculations conducted in this study:

$$RMSE=\sqrt{\frac{\sum_{i=1}^{n}{\left(x_{i}-y_{i}\right)}^{2}}{n}},$$ (17)

$$MAE=\frac{\sum_{i=1}^{m}\left|x_{i}-y_{i}\right|}{n}.$$ (18)

**Fig 6:**
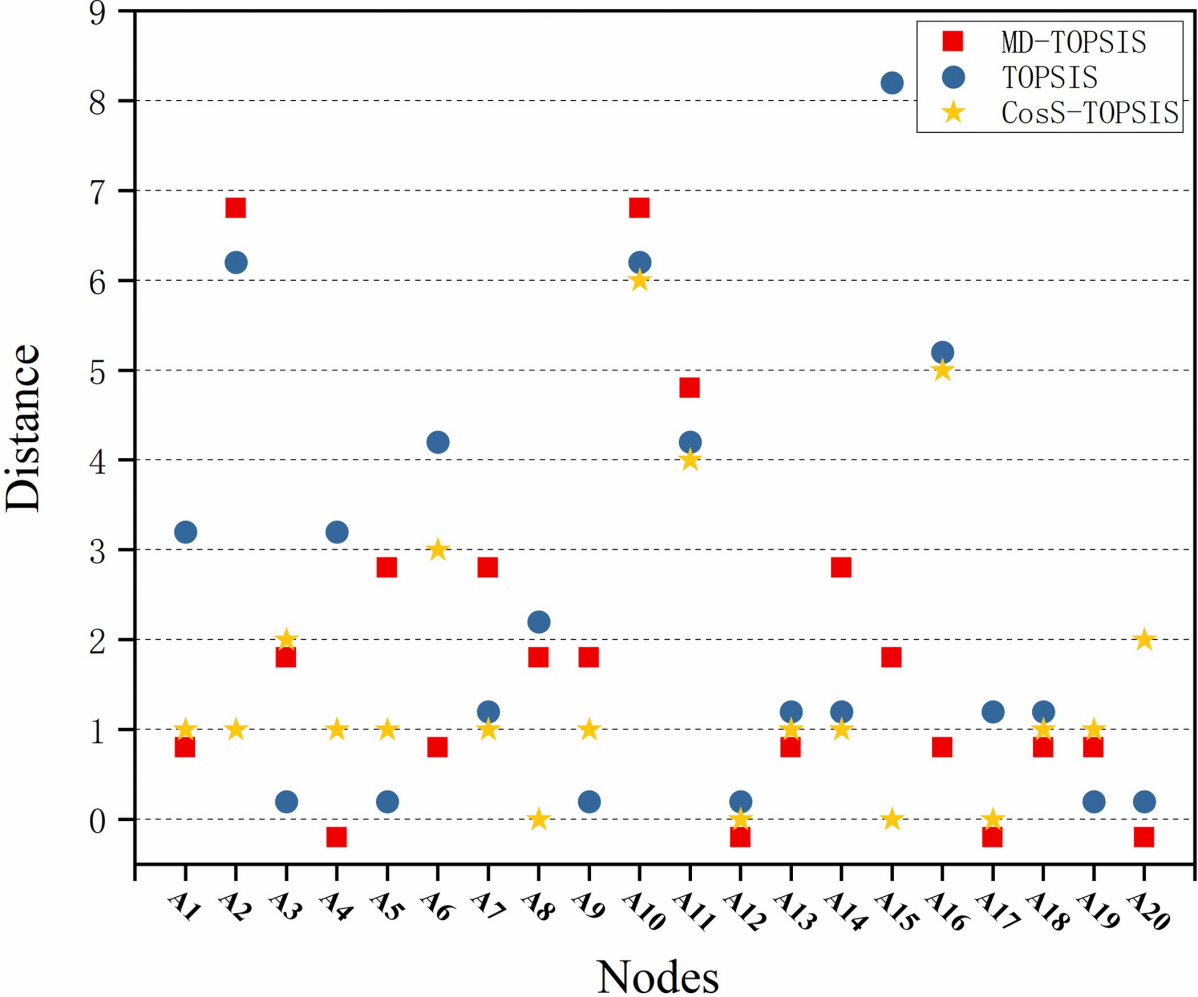
Distance between the results and vulnerability values of cyberspace key terrain assets. The greater the distance, the greater the error. CosS, cosine similarity; MD, Mahalanobis distance; TOPSIS, Technique for Order-of-Preference by Similarity to the Ideal Solution.

RMSE and MAE are also appropriate for assessing the accuracy of each method by taking the results of TOPSIS and improved TOPSIS as the observed values and the system vulnerability values as the actual values. The lower the RMSE value of each method compared with the system fragility value, the higher is the method’s accuracy. Fig 6 shows the distance between the results of each method and the system fragility values.

The information presented in Fig 6 illustrates the difference between the ranking results of the three methods and CVSS 3.1. To address the issue of overlapping and ensure visibility, we vertically adjusted the *Y* values. Specifically, we shifted the value of the MD-TOPSIS method downwards and that of the TOPSIS method upwards.

### Results

We applied CosS-TOPSIS, TOPSIS with an improved Mahalanobis distance (MD-TOPSIS), and the CVSS 3.1-based cyber system fragility value method to identify 20 nodes of an asset layer cyberspace. The results are shown in Table 5.

**Table 5:** Comparison of results of cosine similarity (CosS), Mahalanobis distance (MD), and our improved Technique for Order-of-Preference by Similarity (TOPSIS) methods.

MD-TOPSIS	TOPSIS	CosS-TOPSIS	Vulnerability
A13	A20	A13	A18
A3	A8	A18	A13
A18	A10	A10	A8
A20	A18	A8	A20
A12	A13	A20	A10
A10	A12	A3	A6
A14	A6	A6	A19
A6	A3	A12	A12
A19	A14	A14	A3
A8	A11	A11	A14
A11	A17	A19	A5
A1	A19	A1	A1
A7	A1	A7	A2
A5	A7	A2	A7
A2	A2	A17	A17
A17	A5	A5	A11
A4	A4	A4	A4
A15	A9	A15	A9
A9	A15	A16	A15
A16	A16	A9	A9

*Notes*: Symbols A1–A20 represent 20 nodes in the network space, and the specific corresponding IP addresses are listed in Table 4.

#### Reliability analysis

The experimental results indicate that the multi-attribute decision-class method is reliable. Because the remaining attributes of the cyber node exhibit a strong correlation with node vulnerability (Fig 5), we used the CVSS 3.1 system fragility value method as a reference point to evaluate the reliability of our proposed method. As listed in Table 5, the results of the multi-attribute decision class methods tend to agree with those of the CVSS 3.1 method. The cyber nodes that obtained top rankings using the three multi-attribute decision methods were also ranked higher according to CVSS 3.1. Additionally, the SRC between the TOPSIS methods and CVSS exceeded 0.8, as shown in Fig 6. These findings illustrate the reliability of the model.

#### Variance analysis

Although the results of multi-attribute decision class methods trended consistently, the results of TOPSIS, CosS-TOPSIS, and MD-TOPSIS varied. For instance, the top-ranked nodes in cyberspace were considered as the asset layer of the CKT. Then, the top four network node IPs in the key terrain of this cyberspace identified by our method were A13, A18, A10, and A8; the top four network node IPs in the TOPSIS method were A20, A8, A10, and A18. The top four network node IPs in the MD-TOPSIS method were A13, A3, A18, and A20.

As shown in Table 6, the RMSE between the proposed method and the system vulnerability value was 16% smaller than TOPSIS and 22% smaller than MD-TOPSIS. Furthermore, the MAE of the proposed method was 20% smaller than TOPSIS and 24% smaller than MD-TOPSIS.

**Table 6:** Root mean-square error (RMSE) and mean absolute error (MAE) assessments of the compared methods.

	MD-TOPSIS	TOPSIS	CosS-TOPSIS
**RMSE**	2.93	2.70	**2.28**
**MAE**	2.1	2.0	**1.6**

*Notes*: CosS, cosine similarity; MD, Mahalanobis distance; TOPSIS, Technique for Order-of-Preference by Similarity to the Ideal Solution.

The contemporary TOPSIS method undermined the correlations among multiple cyberspace node attributes, resulting in the failure of positive and negative ideal distances, causing the identified CKT to be biased. Although MD-TOPSIS can reduce the similarity between multiple attributes, the Mahalanobis distance amplifies the effect of irrelevant attributes on the results [22, 23]. Our method reduces both the impact of correlations between multiple attributes on CKT identification and avoids the disadvantages of the Mahalanobis distance.

**Fig 7:**
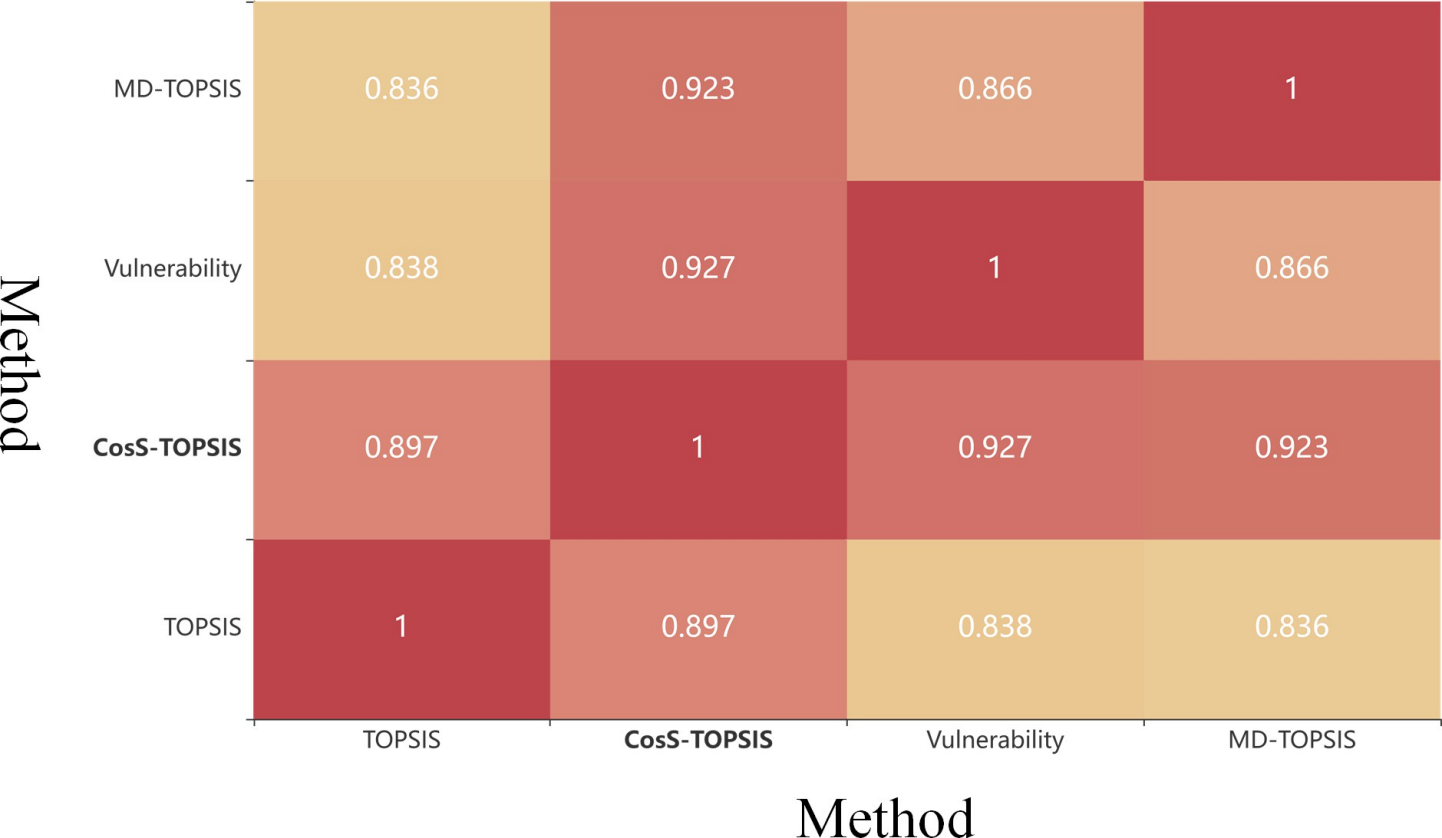
Spearman correlation between methods used to identify the cyberspace key terrain. The color intensity increases as the correlation strength increases. Abbreviations used in the context include CosS for cosine similarity, MD for Mahalanobis distance, and TOPSIS for Technique for Order-of-Preference by Similarity to the Ideal Solution.

As shown in Fig 7, the proposed method was the most similar to the network system fragility value method, indicating that it is more accurate than the others at identifying CKTs. Conversely, as shown in Table 6, our method exhibited the lowest RMSE and MAE values, indicating that the error between the value achieved by our method and the system vulnerability value was the smallest (specifically, the strongest correlation). This indicates that our method is the most reliable when used to explore the topographies of real physical layer CKTs in a given cyberspace to identify vulnerable nodes.

Overall, our approach outperforms current methods and can effectively improve the accuracy of identifying critical CKTs, reduce the possibility of hacking specific overlooked network nodes, and improve the security of the network infrastructure.

### Impact of CAMs on identifying CKTs

The dynamic nature of cyberspace refers to the continuous addition and withdrawal of cyber nodes and the quick update and disappearance of information, which cause weak or drastic changes in the cyberspace terrain. Our work extends the dynamics of cyberspace and argues that these dynamics are not only reflected in the addition and extinction of cyber nodes but are also closely related to CAMs. This dynamic expansion facilitates the ability of cyberspace managers to sense changes in CKTs and promptly adapt their defense strategies.

**Fig 8:**
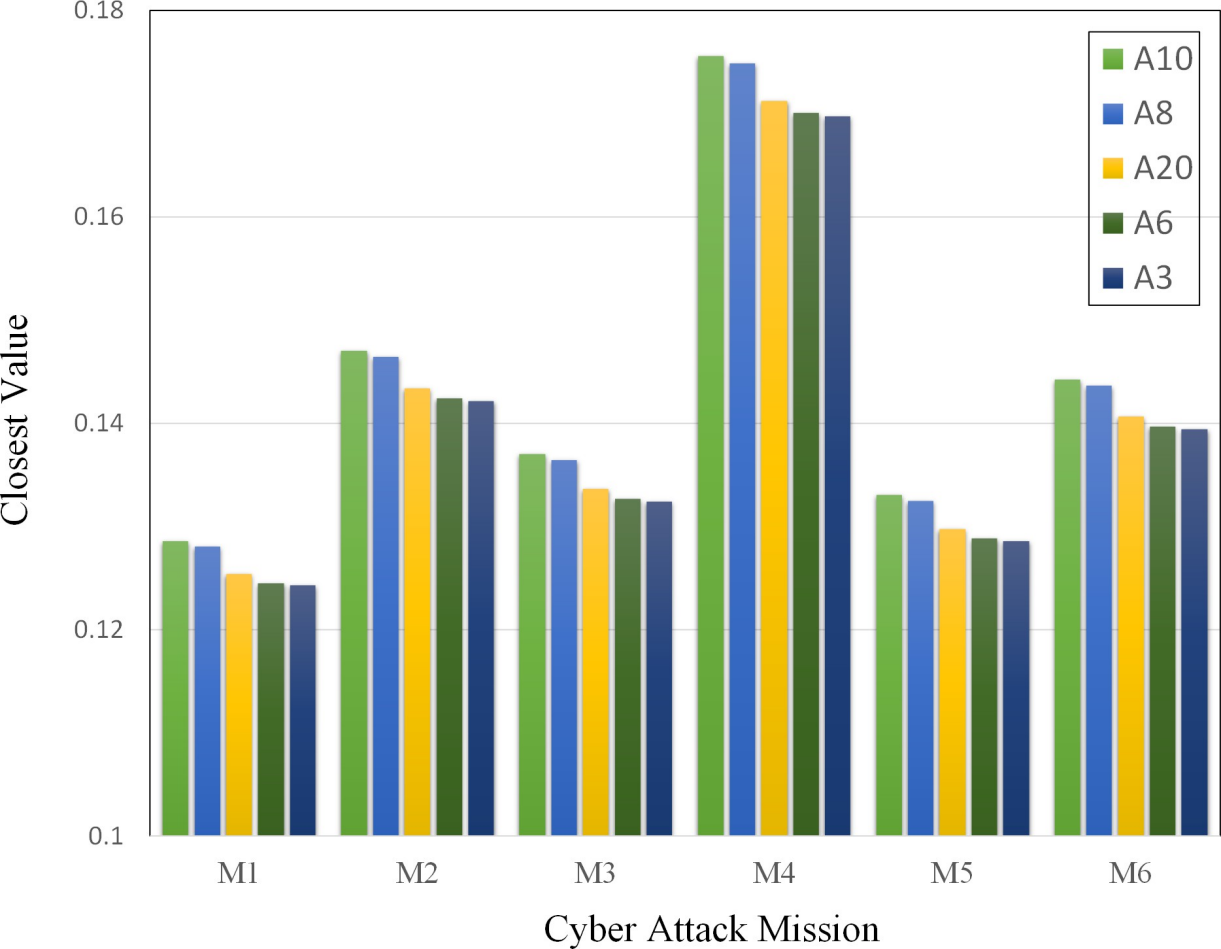
Closest value for different CAMs. M1–M6 represent different network attack tasks, while A10, A8, A20, A6, and A3 represent various cyber nodes.

As shown in Fig 8, the closeness value of node A8 executing the cyberspace attack mission codenamed M4 is smaller than that of node A10 executing the cyberspace attack mission codenamed M3. Table 7 lists the rankings of the cyberspace nodes for different missions. This indicates that CAM has a decisive influence on the identification of critical terrain in cyberspace. The critical terrain changes with the cyberspace attack mission, and the ranking of network nodes under different cyberspace attack missions differs.

**Table 7:** Comparison of results of different mission.

Ranking of cyberspace node
A10(M4)>A8(M2)>A20(M6)>A6(M3)>A3(M5)
A8(M4)>A10(M6)>A20(M2)>A3(M6)>A6(M3)
A3(M4)>A10(M2)>A8(M2)>A20(M3)>A6(M1)

## Conclusion

Cyberspace has become increasingly prosperous; therefore, tapping into its weaknesses to provide decision support for cyber defense has become a significant problem that needs to be solved. In this study, we proposed CosS-TOPSIS for modeling the key terrain in cyberspace, and the dynamics of cyberspace were extended based on cyberspace attack mission weights. Our experiments showed that, in comparison with the TOPSIS method, the accuracy of the proposed method in identifying the asset layer CKT improved by 8.9%; meanwhile, it was proven that a cyberspace attack mission is an essential factor affecting the cyberspace key terrain modeling.

Our approach has significant application potential in the field of cyberspace security. Within the physical layer CKT, each CKT represents one or more assets. Based on the multi-attribute data of network nodes, cyberspace managers can utilize our method to expedite the identification of nodes with increased risk. Subsequently, they can monitor changes, address vulnerabilities, and improve protection measures. Our method effectively screens significant nodes within potentially extensive physical layers of CKTs, providing valuable assistance to decision makers in identifying and reacting to risks and vulnerabilities. This capability clearly holds implications for enhancing cyberspace defense.

However, our method has the following limitations. When constructing weights, expert decisions are required; this can easily influence objectivity. Furthermore, the cyberspace attack mission is random, and cyberspace attack missions with different number of tactics are not considered in our work. Similarly, given the complexity of cyberspace, the scope of our method is limited to the asset layer of cyberspace, and the influence of the cyber decision, command, and geospatial layers on CKT is yet to be considered. In future work, we plan to introduce entropy and other methods to reduce the subjectivity of the assignment, use more elemental layers in cyberspace in the criterion layer, and analyze the influence of each elemental layer on the cyberspace key terrain.
